# Supplementary material for: T-Cell Infiltration and Clonality May Identify Distinct Survival Groups in Colorectal Cancer: Development and Validation of a Prognostic Model Based on The Cancer Genome Atlas (TCGA) and Clinical Proteomic Tumor Analysis Consortium (CPTAC)
Source: Cancers (Basel). 2022 Nov 29;14(23):5883. doi: 10.3390/cancers14235883 (PMC9740634; doi:10.3390/cancers14235883)
Supplement: Supplementary file 1 [file cancers-14-05883-s001.zip › Campana et al SuppTable 1.pdf]

**Covariates retained in the final model after deleting factors**	**Coefficient**	**Standard Error**	**Wald Z**	**P**
Age	0.03297*	0.007439	4.433	<.0001
Stage				
Localised	-1.34168	0.200989	-6.675	<.0001
Advanced	*reference*			
TIL/Tc infiltration grade				
Low	0.55129	0.219018	2.517	0.01183
High	*reference*			
Very high	1.43771	0.275678	5.215	<.0001
TIL/Tc clonality	-1.86914*	0.628800	-2.973	0.002953
**Covariates deleted with backward selection**	**Residual**	**Degrees of freedom**	**P**	**AIC**
Sideness (left vs right)	5.90	4	0.4345	-2.14
Sex	2.88	6	0.8242	-9.12
TIL/Tc diversity	5.97	7	0.5429	-8.03
Total SNSs	6.52	9	0.6867	-11.48
Second dominant SNS signature	5.86	4	0.2095	-2.14
MANTIS-MSI score	5.86	5	0.3196	-4.14
IFNγ mRNA z-score	6.08	8	0.6867	-11.48

Supplementary Table 1. Covariates deleted after backward selection
Summary of the Cox regression model analysis for colorectal cancer TCGA cohort. Disease stage is classified according to the TNM annotation in the TCGA clinical information file (localised is stage I, II and advanced is III and IV). Coefficient is ln(hazard ratio), TCGA is The Cancer Genome Atlas, TIL/Tc is tumour infiltrating T cells, SNS is single nucleotide substitution, TCR is T cell receptor, AIC is Akaike information criterion; * age and TIL/Tc clonality are retained as continuous variables and the coefficient value refers to unitary increments of the covariate.
