# Supplementary material for: T-Cell Infiltration and Clonality May Identify Distinct Survival Groups in Colorectal Cancer: Development and Validation of a Prognostic Model Based on The Cancer Genome Atlas (TCGA) and Clinical Proteomic Tumor Analysis Consortium (CPTAC)
Source: Cancers (Basel). 2022 Nov 29;14(23):5883. doi: 10.3390/cancers14235883 (PMC9740634; doi:10.3390/cancers14235883)
Supplement: Supplementary file 1 [file cancers-14-05883-s001.zip › Campana et al SuppTable 2.pdf]

Covariate	coefficient	HR	SE (coefficient)	Z	P
MANTIS-MSI score	0.8525	2.3455	0.9953	0.857	0.392

Supplementary Table 2. Prognostic impact of MANTIS-MSI score on survival in the CRC cancers with “very high” TIL/Tc infiltration.
Results of univariate Cox regression analysis testing the relationship between MANTIS-MSI score and overall survival; likelihood ratio test=0.67 on 1 degree of freedom. HR=hazard ratio; SE=standard error.
