# Supplementary material for: T-Cell Infiltration and Clonality May Identify Distinct Survival Groups in Colorectal Cancer: Development and Validation of a Prognostic Model Based on The Cancer Genome Atlas (TCGA) and Clinical Proteomic Tumor Analysis Consortium (CPTAC)
Source: Cancers (Basel). 2022 Nov 29;14(23):5883. doi: 10.3390/cancers14235883 (PMC9740634; doi:10.3390/cancers14235883)
Supplement: Supplementary file 1 [file cancers-14-05883-s001.zip › Campana et al SuppTable 3.pdf]

TCGA Participant ID	AJCC Stage
TCGA-A6-2686-01A	Stage IIA
TCGA-AA-A01P-01A	Stage III
TCGA-AY-6196-01A	Stage IIIC
TCGA-CA-6717-01A	Stage IIA
TCGA-CA-6718-01A	Stage IIA
TCGA-D5-6534-01A	Stage IIA
TCGA-D5-6928-01A	Stage IIA
TCGA-F4-6570-01A	Stage IIA
TCGA-G4-6321-01A	Stage III
TCGA-G4-6628-01A	Stage I
TCGA-QG-A5Z2-01A	Stage I

Supplementary Table 3. Clinical stage of the BRAF mutated CRC cancers with very high TIL/Tc infiltration. AJCC clinical stage of the TCGA CRC cancers with BRAF mutation, as reported in the TCGA MAF file and clinical metafile.
