# Supplementary material for: T-Cell Infiltration and Clonality May Identify Distinct Survival Groups in Colorectal Cancer: Development and Validation of a Prognostic Model Based on The Cancer Genome Atlas (TCGA) and Clinical Proteomic Tumor Analysis Consortium (CPTAC)
Source: Cancers (Basel). 2022 Nov 29;14(23):5883. doi: 10.3390/cancers14235883 (PMC9740634; doi:10.3390/cancers14235883)
Supplement: Supplementary file 1 [file cancers-14-05883-s001.zip › SuppFigure8.PDF]

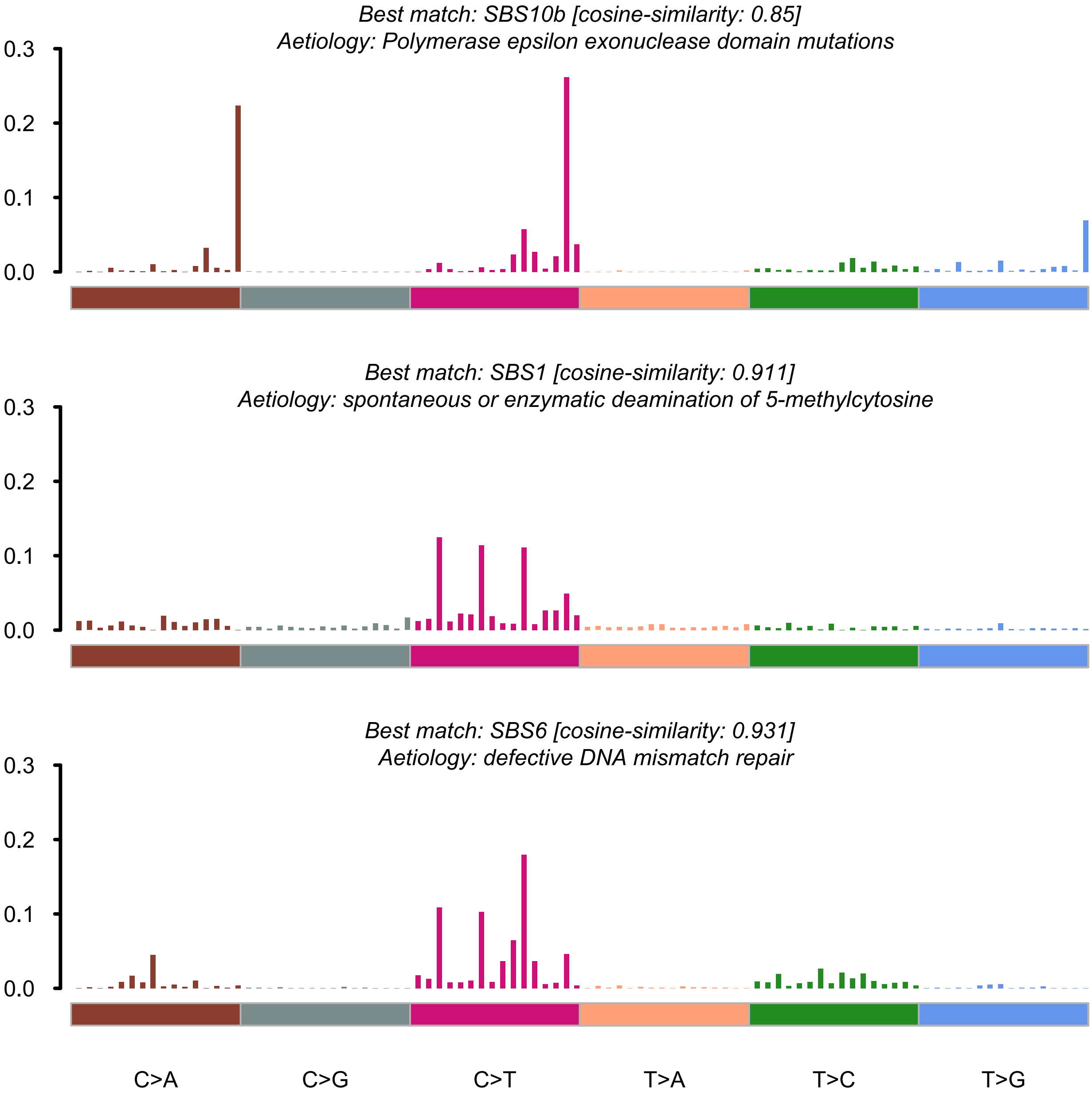
Best match: SBS10b [cosine-similarity: 0.85]
Aetiology: Polymerase epsilon exonuclease domain mutations
Best match: SBS1 [cosine-similarity: 0.911]
Aetiology: spontaneous or enzymatic deamination of 5-methylcytosine
Best match: SBS6 [cosine-similarity: 0.931]
Aetiology: defective DNA mismatch repair
0.3
0.2
0.1
0.0
C>A
C>G
C>T
T>A
T>C
T>G
