# Supplementary figures and images for: T-Cell Infiltration and Clonality May Identify Distinct Survival Groups in Colorectal Cancer: Development and Validation of a Prognostic Model Based on The Cancer Genome Atlas (TCGA) and Clinical Proteomic Tumor Analysis Consortium (CPTAC)
Source: Cancers (Basel). 2022 Nov 29;14(23):5883. doi: 10.3390/cancers14235883 (PMC9740634; doi:10.3390/cancers14235883)

MANTIS MSI score

10000  
9000  
8000  
7000  
6000  
5000  
4000  
3000  
2000  
1000  
0

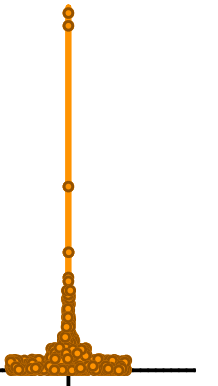

Supplement: Supplementary file 1 [file cancers-14-05883-s001.zip › SuppFigure1.pdf]

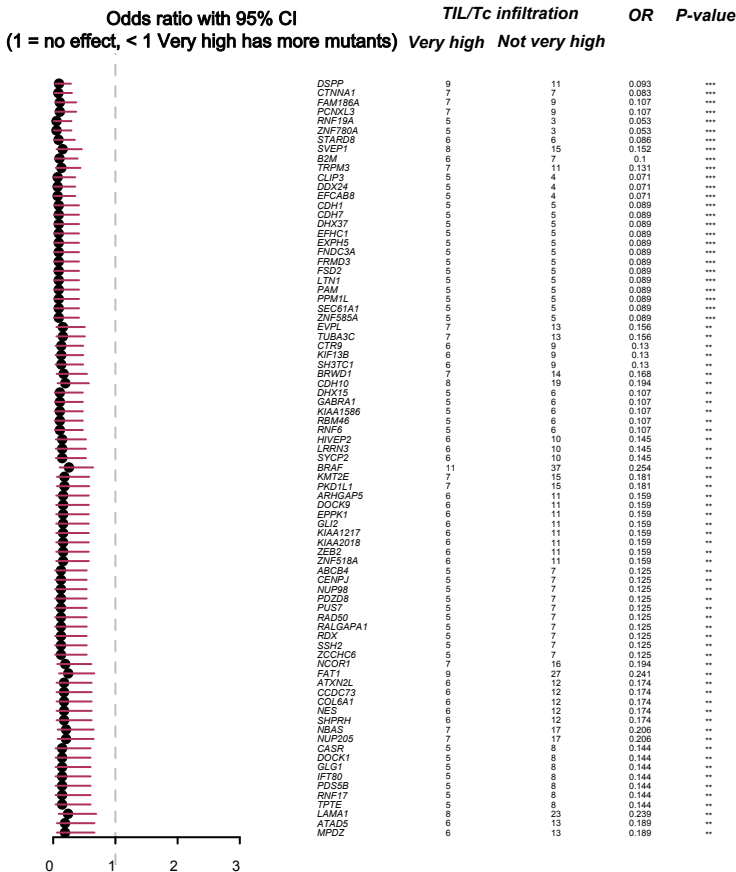

Supplement: Supplementary file 1 [file cancers-14-05883-s001.zip › SuppFigure10.PDF]

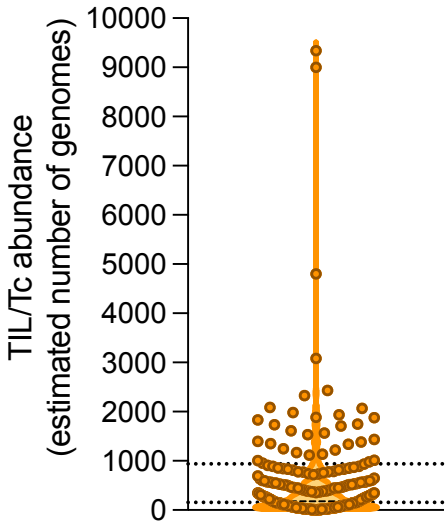

Supplement: Supplementary file 1 [file cancers-14-05883-s001.zip › SuppFigure2.pdf]

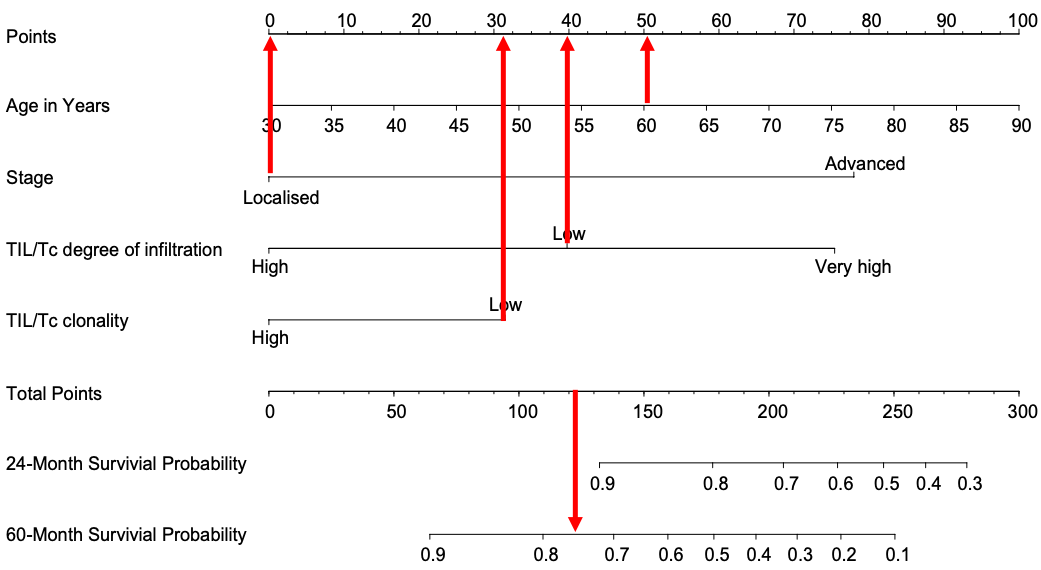

Supplement: Supplementary file 1 [file cancers-14-05883-s001.zip › SuppFigure3.png]

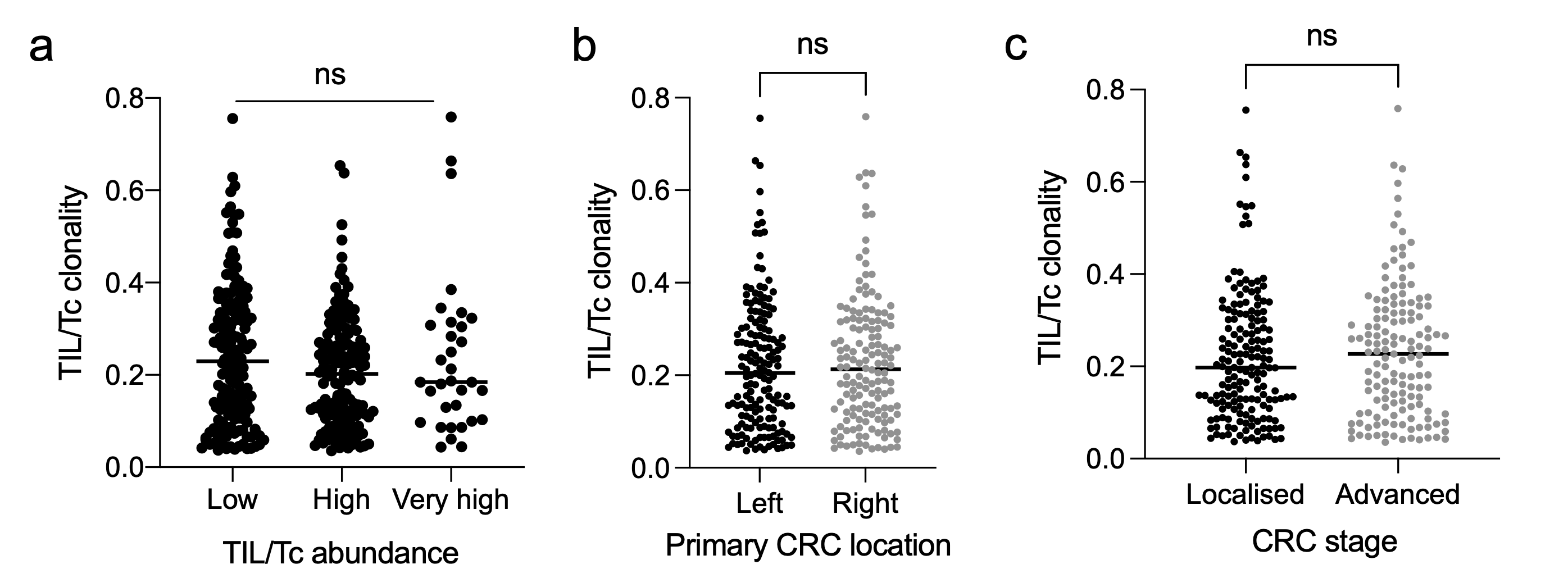

Supplement: Supplementary file 1 [file cancers-14-05883-s001.zip › SuppFigure4.png]

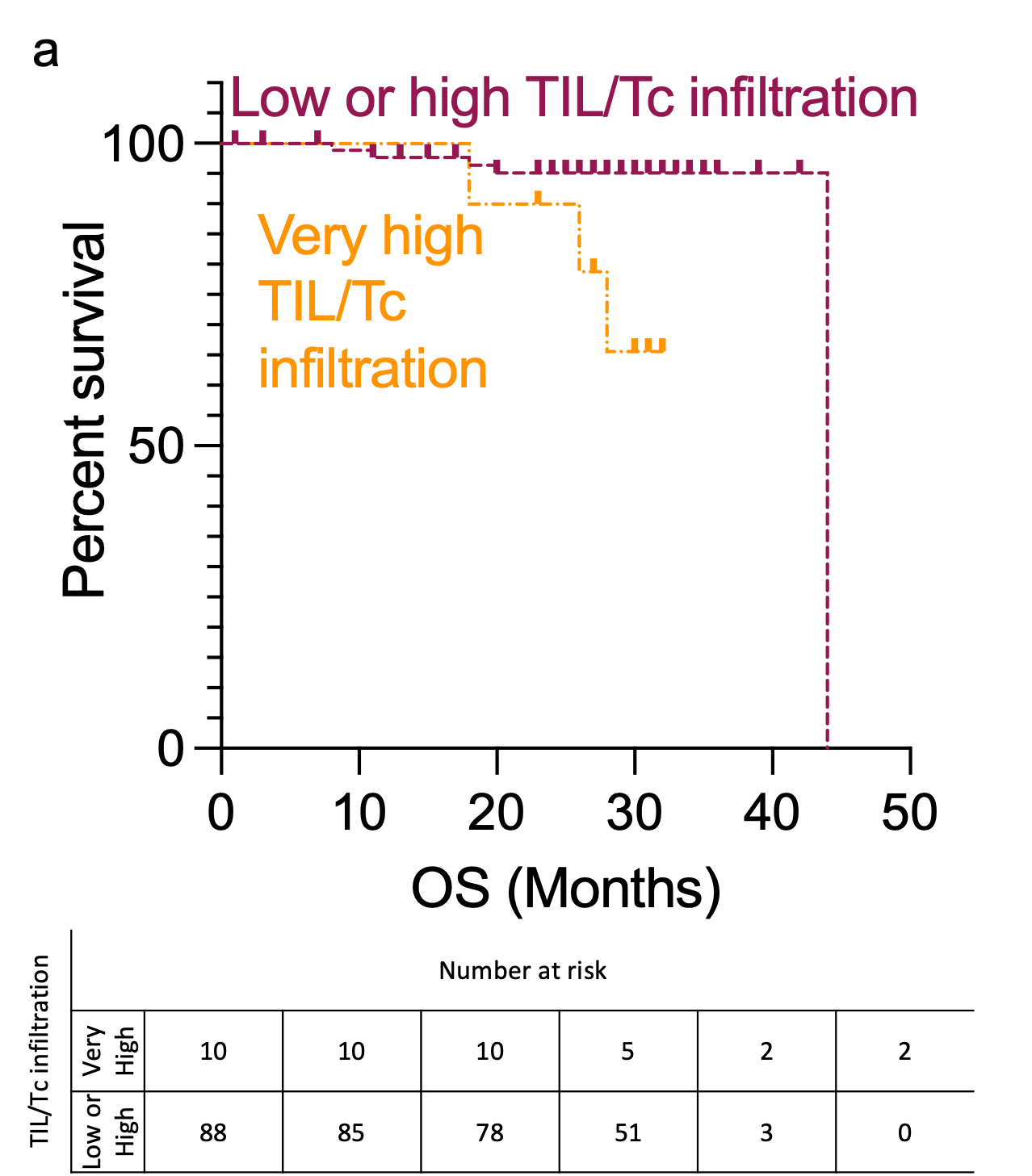

Supplement: Supplementary file 1 [file cancers-14-05883-s001.zip › SuppFigure5.png]

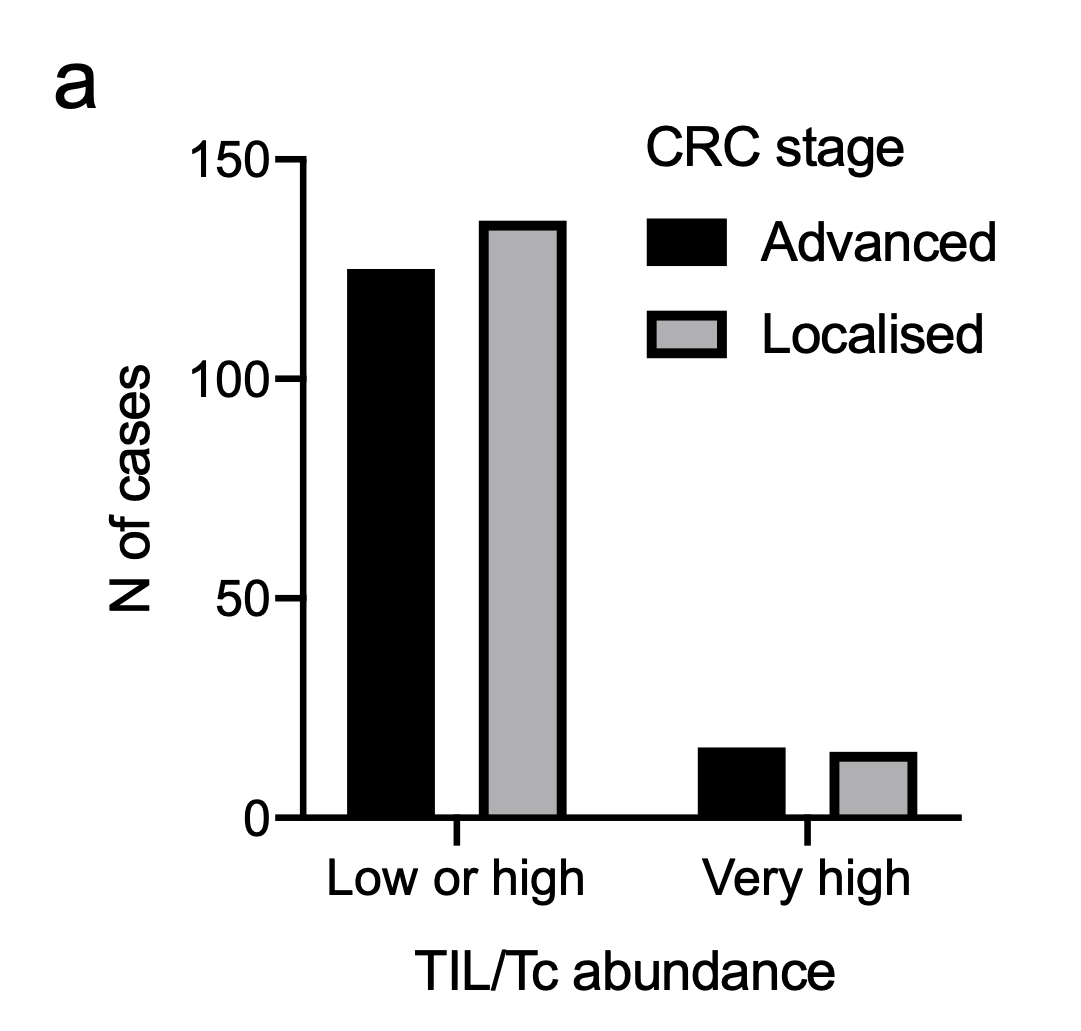

Supplement: Supplementary file 1 [file cancers-14-05883-s001.zip › SuppFigure6.png]

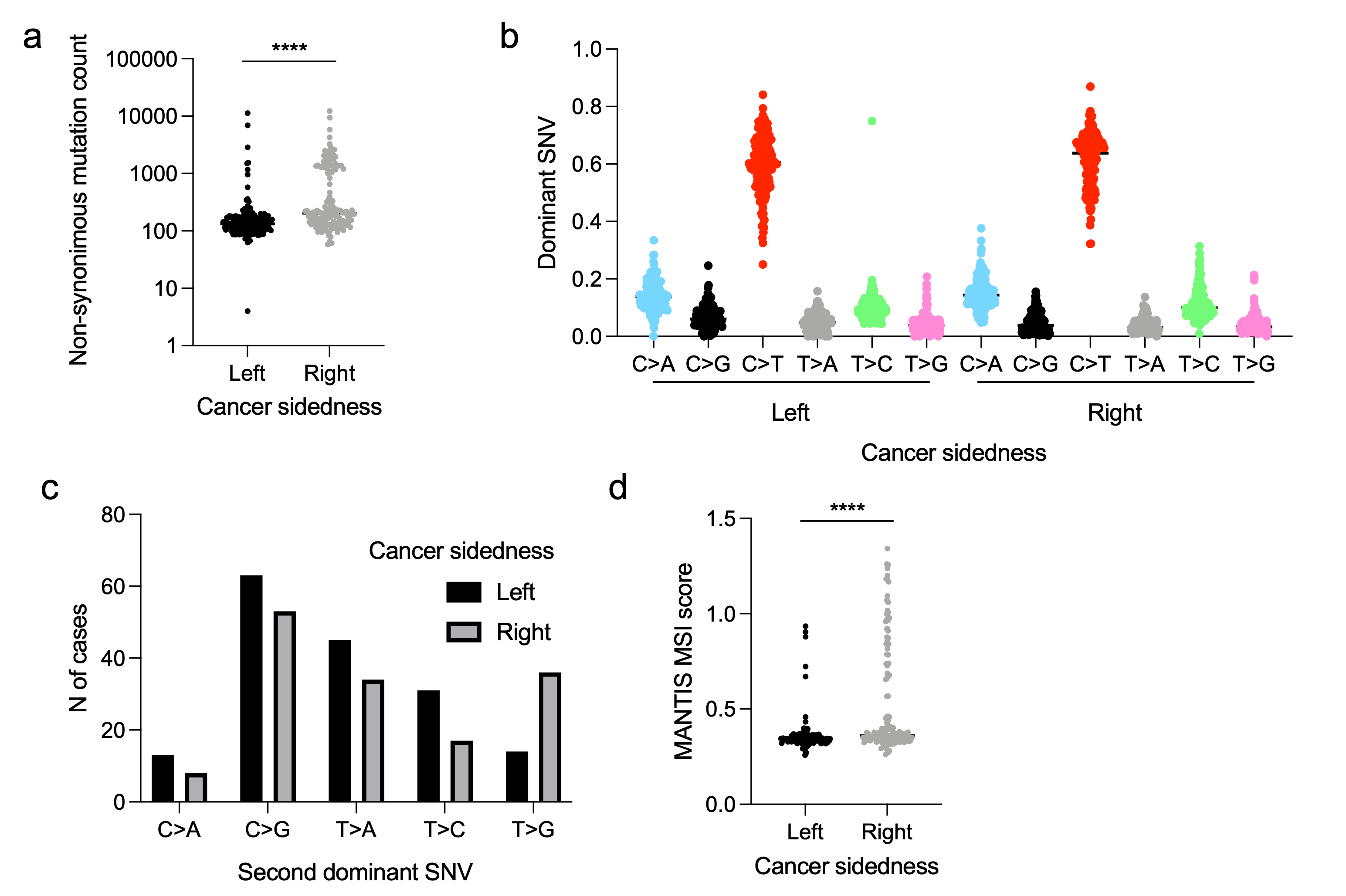

Supplement: Supplementary file 1 [file cancers-14-05883-s001.zip › SuppFigure7.png]
